# Removal of Per- and Polyfluoroalkyl Substances from Contaminated Groundwater using Cationic Metal−Organic Frameworks

**DOI:** 10.1063/4.0001172

**Published:** 2025-10-27

**Authors:** Arefeh Mirsharifian, Mario Wriedt

**Affiliations:** University of Texas at Dallas, Richardson, Texas, USA

## Abstract

Per- and polyfluoroalkyl substances (PFAS) are highly persistent and toxic environmental contaminants frequently detected in water sources, posing serious threats to both human health and ecosystems. Conventional treatment methods, such as activated carbon and ion exchange resins, often show limited effectiveness across the diverse range of PFAS compounds. Cationic metal–organic frameworks (MOFs) have emerged as promising adsorbents for PFAS removal due to their crystallinity, high surface area, and strong electrostatic interactions with anionic species. In this study, we evaluated the performance of selected cationic MOFs for the adsorption of PFAS mixtures from aqueous systems.

Our results demonstrated removal efficiencies of up to 99.9%, underscoring the strong potential of these materials for effective PFAS remediation. These findings highlight the promise of cationic MOFs as viable and efficient adsorbents to help mitigate the growing environmental challenge posed by PFAS contamination in water.

